# P-1162. Colonization Rates and Antimicrobial Susceptibility of *Mycoplasma hominis* and *Ureaplasma urealyticum* in Adolescent Girls: Comparison with Young Adults

**DOI:** 10.1093/ofid/ofae631.1348

**Published:** 2025-01-29

**Authors:** Soo-Han Choi, Soo-Han Choi

**Affiliations:** Department of Pediatrics, Hallym University Dongtan Sacred Heart Hospital, Hallym University College of Medicine, Hwaseong, Kyonggi-do, Republic of Korea; Pusan National University School of Medicine, Busan, Not Applicable, Republic of Korea

## Abstract

**Background:**

Sexually transmitted infection rates are increasing among adolescents and young adults and remain a serious health problem worldwide. *Mycoplasma hominis* and *Ureaplasma* species are sexually transmitted and considered etiologic agents causing various urogenital diseases. This study aimed to estimate the prevalence and antimicrobial susceptibility of *M. hominis* and *U. urealyticum* in adolescent girls and to compare them with young women.

**Methods:**

We evaluated the results of *M. hominis* and *U. urealyticum* isolated from vaginal swab culture between January 2011 and August 2023. The data were selected from young women aged 12-26 and analyzed by dividing the two groups into adolescents (12-18 years) and young adults (19-26 years).

**Results:**

Among 66 adolescent girls, 58 (87.9%) were positive for mycoplasma culture. Twenty-nine (43.9%) had both *M. hominis* and *U. urealyticum*. Compared with the young adult group, colonization rates of *M. hominis* (45.5% [30/66] *vs*. 21.7% [64/295], *P*=0.0002) and *U. urealyticum* (86.4% [57/66] *vs*. 79.4% [219/295], *P*=0.0373) were significantly higher in the adolescent group. As age increased, colonization rates of *M. hominis* (rho = -0.6703; 95% confidence interval [CI], -0.8840 to -0.2248) and *U. urealyticum* (rho = -0.5993; 95% CI, -0.8550 to -0.1091) tended to decrease. Macrolides (azithromycin, clarithromycin, and erythromycin) susceptibility rate of *M. hominis* was less than 10% in both groups. Of *M. hominis* isolates from adolescents, susceptibility rates to azithromycin, clarithromycin, and erythromycin were 0.0%, 6.7%, and 0.0%, respectively. Ninety percent and 76.7% of *M. hominis* isolates from adolescents were susceptible to doxycycline and tetracycline. Compared with the young adult group, the macrolides susceptibility rate of *U. urealyticum* was significantly lower in the adolescent group: azithromycin (36.8% *vs*. 53.4%, *P*=0.0368), clarithromycin (47.4% *vs*. 68.9%, *P*=0.0031), and erythromycin (38.6% *vs*. 54.3%, *P*=0.038). Susceptibility rates of *U. urealyticum* to doxycycline and tetracycline were 94.7% and 87.7% in the adolescent group.

**Conclusion:**

Compared with young adults, higher rates of colonization and resistance to macrolides for both *M. hominis* and *U. urealyticum* were more frequently observed in adolescent girls.

**Disclosures:**

**All Authors**: No reported disclosures

